# Supplementary figures and images for: Alpha-Melanocyte-Stimulating Hormone Maintains Retinal Homeostasis after Ischemia/Reperfusion
Source: Biomolecules. 2024 Apr 27;14(5):525. doi: 10.3390/biom14050525 (PMC11118772; doi:10.3390/biom14050525)

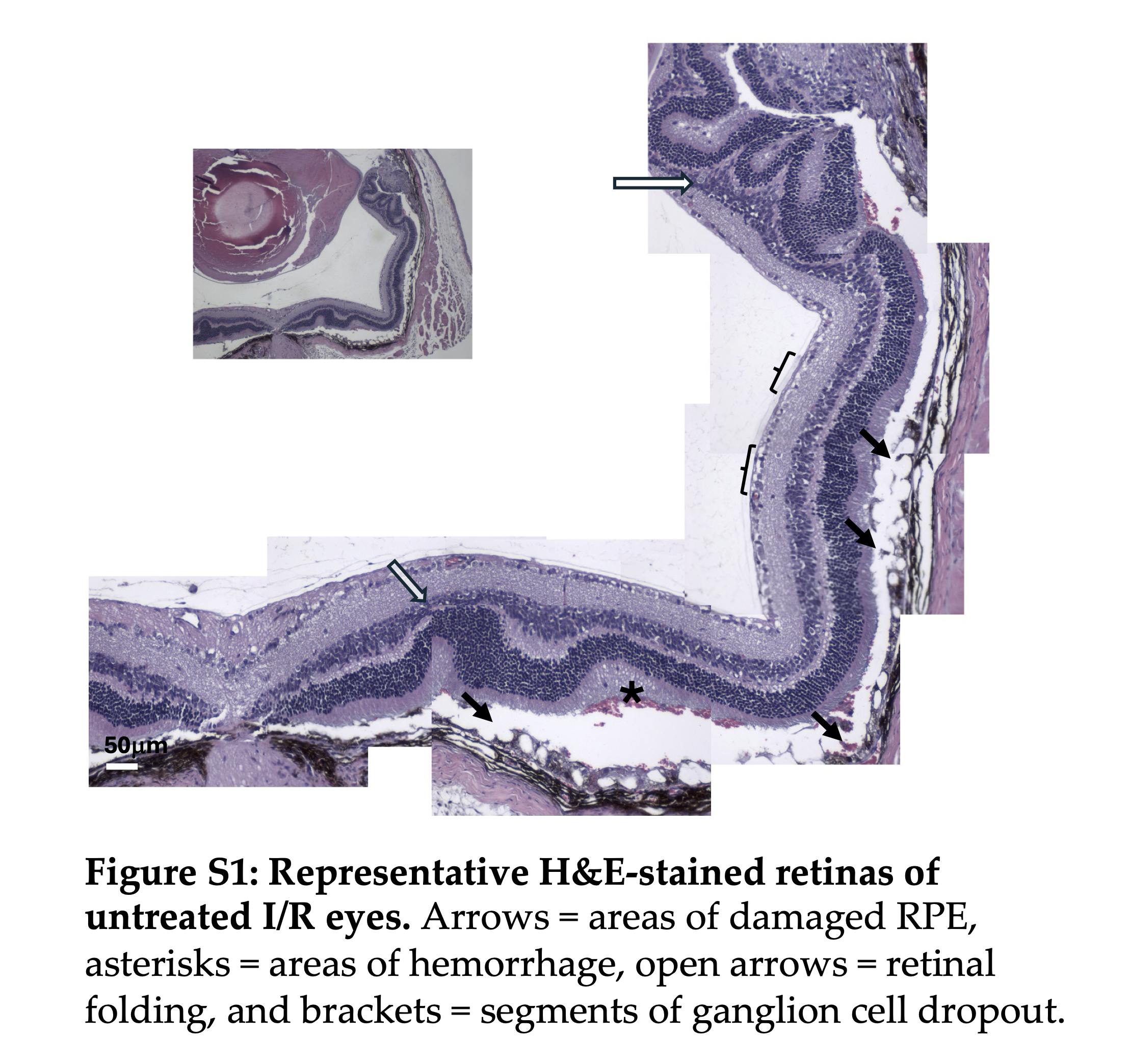

Supplement: Supplementary file 1 [file biomolecules-14-00525-s001.zip › Figure S1.tiff]
